# Bacille Calmette–Guerin Complications in Newly Described Primary Immunodeficiency Diseases: 2010–2017

**DOI:** 10.3389/fimmu.2018.01423

**Published:** 2018-06-22

**Authors:** Cristiane de Jesus Nunes-Santos, Sergio D. Rosenzweig

**Affiliations:** ^1^Faculdade de Medicina, Instituto da Crianca, Universidade de São Paulo, São Paulo, Brazil; ^2^Immunology Service, Department of Laboratory Medicine, NIH Clinical Center, National Institutes of Health (NIH), Bethesda, MD, United States

**Keywords:** mycobacteria, bacille Calmette–Guerin, primary immunodeficiency, live vaccines, complications, adverse reactions

## Abstract

Bacille Calmette–Guerin (BCG) vaccine is widely used as a prevention strategy against tuberculosis. BCG is a live vaccine, usually given early in life in most countries. While safe to most recipients, it poses a risk to immunocompromised patients. Several primary immunodeficiency diseases (PIDD) have been classically associated with complications related to BCG vaccine. However, a number of new inborn errors of immunity have been described lately in which little is known about adverse reactions following BCG vaccination. The aim of this review is to summarize the existing data on BCG-related complications in patients diagnosed with PIDD described since 2010. When BCG vaccination status or complications were not specifically addressed in those manuscripts, we directly contacted the corresponding authors for further clarification. We also analyzed data on other mycobacterial infections in these patients. Based on our analysis, around 8% of patients with gain-of-function mutations in *STAT1* had mycobacterial infections, including localized complications in 3 and disseminated disease in 4 out of 19 BCG-vaccinated patients. Localized BCG reactions were also frequent in activated PI3Kδ syndrome type 1 (3/10) and type 2 (2/18) vaccinated children. Also, of note, no BCG-related complications have been described in either CTLA4 or LRBA protein-deficient patients; and not enough information on BCG-vaccinated NFKB1 or NFKB2-deficient patients was available to drive any conclusions about these diseases. Despite the high prevalence of environmental mycobacterial infections in GATA2-deficient patients, only one case of BCG reaction has been reported in a patient who developed disseminated disease. In conclusion, BCG complications could be expected in some particular, recently described PIDD and it remains a preventable risk factor for pediatric PIDD patients.

## Introduction

Based on the World Health Organization (WHO) Global Tuberculosis Report, tuberculosis remains a public health global problem: it is the ninth leading cause of death worldwide, and the leading cause of death from a single pathogen (1). Bacille Calmette–Guerin (BCG) vaccine is widely used as a prevention strategy against tuberculosis. The BCG vaccine was developed between 1908 and 1921 by Albert Calmette and Camille Guerín in France by culturing and attenuating a live strain of *Mycobacterium bovis*. BCG was first administered to humans in 1921 and has been used for more than 95 years until now (2).

Vaccination policies vary around the world, linked mostly to tuberculosis disease prevalence. While tuberculosis endemic areas (mainly in developing countries) adopt universal vaccination, tuberculosis low-prevalence countries either restrict BCG vaccine to high-risk groups or choose not to administer it at all (3). Controversies surrounding the vaccine’s efficacy account for variations in vaccination policies. While BCG vaccine is believed to provide a somehow consistent protection against severe forms of tuberculosis (i.e., miliary, meningeal) in childhood, most adult individuals remain susceptible to pulmonary tuberculosis despite vaccination (4). As previous exposure to nontuberculous mycobacteria (NTM) seems to influence vaccine efficacy, and to assure full coverage, BCG is usually given right after birth in the first months of life (5).

Even in the context of its questionable efficacy, BCG vaccine is considered safe in immunocompetent subjects (6). However, being a live vaccine, it can result in serious illness or even fatal disease in immunocompromised hosts (7). For instance, WHO guidelines recommend holding BCG vaccination in high-risk infants until assessment of HIV status (8). Patients with primary immunodeficiency diseases (PIDD) are at equal or even greater risk of complications and represent a challenging group regarding live vaccines in general and BCG vaccination in particular (7, 9).

Primary immunodeficiency diseases are inborn errors of immunity which commonly lead to increased susceptibility to infection (10). Defects impairing cellular immunity, phagocytic function, and interferon-γ-mediated immunity have been classically associated with BCG vaccine complications (11). The advent of next-generation sequencing technology boosted discoveries in the field of PIDD. To date, 354 different disorders affecting 344 genes have been described, nearly one-third of them after 2010 (12). Meanwhile, the potential impact of recently described PIDD on BCG immune response remains blurry, as our understanding of natural history and detailed molecular mechanisms of these defects are still limited and continuously expanding.

The aim of this review is to update and summarize the published data on BCG-related complications in patients diagnosed with PIDD described after 2010. We arbitrarily selected defects affecting either innate (i.e., monocytes, macrophages, or dendritic cells) or adaptive (i.e., T cells) arms of antimycobacterial immunity that had been described in at least 20 patients or 10 unrelated kindreds. Diseases unequivocally classified as having Mendelian susceptibility to mycobacterial diseases (MSMD), were not reanalyzed in this review. Finally, 18 genetic defects or allelic variants associated with particular PIDD were analyzed. Considering the timing of vaccine administration, most patients’ PIDD were undiagnosed when vaccinated. As BCG vaccination history is not described in all publications and many reports come from countries where BCG vaccine is not universally applied, we also collected data on any mycobacterial infection, highlighting the occurrence of weakly virulent strains. For those reports where BCG vaccination status was not specifically described, we directly contacted the corresponding author/s for further clarification in terms of patients’ BCG vaccination status and complications associated with it. The overall retrieve rate for extra information requests was 17%, range 0.8–100%, depending on the specific PIDD analyzed (Table 1).

**Table 1:** Retrieval rate of BCG vaccination status among patients with recently described PIDD.

PIDD	Published cases	BCG vaccination status retrieval
GOF mutations in ***STAT1***		
STAT1	348	43 (12%)
GOF mutations in ***STAT3***		
STAT3	31	7 (23%)
**Activated PI3Kδ syndrome type 1 and 2**		
PIK3CD	158	40 (25%)
PIK3R1	64	49 (77%)
**Defects affecting the NF-κB pathway**		
NFKB1	56	15 (27%)
NFKB2 (DN and GOF)	29	11 (38%)
CBM complex^a^		
CARD11 AR LOF	5	2 (40%)
CARD11 GOF	9	9 (100%)
CARD11 DN/LOF	12	4 (33%)
BCL10	1	–
MALT1	6	4 (67%)
**CTLA-4 and LRBA deficiencies**		
CTLA4	74	14 (19%)
LRBA	108	21 (19%)
**GATA2 deficiency**		
GATA2	357	3 (0.8%)
**CARMIL2 deficiency**		
CARMIL2	21	4 (19%)
**PGM3 deficiency**		
PGM3	37	4 (11%)
**TTC7A deficiency**		
TTC7A	53	–
	1,369	230 (17%)

*^a^For the purpose of the inclusion criteria, all members of the CBM complex were considered as a group*.

*AR, autosomal recessive; BCG, Bacille Calmette–Guerin; DN, dominant negative; GOF, gain-of-function; LOF, loss-of-function; PIDD, primary immunodeficiency diseases; TB, tuberculosis; STAT, signal transducer and activator of transcription; CARD, caspase activation and recruitment domain; BCL10, B-cell leukemia/lymphoma 10; MALT1, mucosa-associated lymphoid tissue lymphoma translocation gene 1; CTLA-4, cytotoxic T lymphocyte antigen-4; LRBA, lipopolysaccharide-responsive Beige-like anchor protein; PGM3, phosphoglucomutase 3; PIK3CD, phosphoinositide kinase 3 catalytic unit delta; PIK3R1, phosphoinositide kinase 3 regulatory subunit 1; NFKB, nuclear factor kappa-light-enhancer of activated B cells; CBM, CARD11, BCL10, MALT1 complex; CARMIL2, capping protein regulator and myosin 1 linker 2; TTC7A, tetratricopeptide repeat domain 7A*.

## Gain-of-Function (GOF) Mutations in Signal Transducer and Activator of Transcription 1 (*STAT1*)

STAT1 is a transcription factor involved in several cytokine-dependent signaling pathways, notably IFN-α/β, IFN-γ, and IL-27 (13, 14). Both biallelic and heterozygous loss-of-function (LOF) mutations of *STAT1* have been previously described and associated with either susceptibility to intracellular bacterial/viral infections, or Mendelian susceptibility to mycobacterial disease, respectively (15–17). In 2011, Liu et al. and van de Veerdonk et al. demonstrated that an enhanced STAT1 activity, as a result of heterozygous GOF mutations of STAT1, was also deleterious, presenting with a chronic mucocutaneous candidiasis (CMC) phenotype due to impaired IL-17 immunity (18, 19). Afterward, several studies confirmed that these mutations caused a delay in nuclear dephosphorylation of activated STAT1 resulting in reduced production of Th17 cells (20–22), which are pivotal for candida-specific immune responses (23).

While CMC is still the hallmark of this disease—it was present in 98% of patients from an international cohort of 274 STAT1 GOF patients (24)—as more patients were described, the phenotype was proven to be much more diverse than initially assumed (22, 24). These patients are at higher risk of other infections (bacterial/viral/fungal) (25–27), autoimmunity (28, 29), aneurisms (30, 31), and malignancies (32, 33). Many of these manifestations cannot be solely explained by a reduction of Th17 cells, implying that excessive activation of STAT1 potentially impairs immunity through other mechanisms not yet fully understood (29, 34, 35).

Twenty-seven reports of mycobacterial infections could be retrieved from more than 350 published STAT1 GOF patients (20, 24, 25, 27, 29, 31, 33, 34, 36–40). Vaccination status was not addressed in most descriptions (3, 18–22, 24–34, 36–39, 41–72). We were able to retrieve BCG vaccination history from 43 patients, of whom 19 received the vaccine (Tables 1 and 2). BCG complications were seen in seven patients. Three patients had local reactions [(29, 37, 38), and Acknowledgments] and four experienced disseminated disease (24, 25, 31). In addition to BCG, other mycobacteria also seem to threaten a subset of these patients (Table 2). Disseminated disease was observed in eight more patients (six cases of *Mycobacterium tuberculosis* and two of NTM) (24, 31, 34, 38, 39), four patients had pulmonary infections caused by environmental mycobacteria (24, 29, 33), and two patients had lymphadenitis caused by *Mycobacterium fortuitum* (27, 37). *M. tuberculosis* caused pulmonary disease in five patients (24, 36, 37) and extrapulmonary localized disease in one patient (20).

**Table 2:** BCG and other mycobacterial complications in recently described PIDD.

PIDD	BCG vaccinated^a^	BCG reaction		NTM	Pulmonary TB	Extrapulmonary TB		Total^b^	References
		Localized	Disseminated			Localized	Disseminated		
STAT1 GOF	19/43	3	4	8 (2 pulmonary *M. avium;* 1 disseminated *M. avium;* 2 *M. fortuitum* lymphadenitis; 1 disseminated *M. genavense;* and 2 lung NTM infection)	5	1	6	348	(3, 18–22, 24–34, 36–39, 41–72)
STAT3 GOF	4/7	–	–	1 (disseminated *M. avium*)	–	–	–	31	(78–82, 84–88)
PIK3CD	10/40	3	–	–	–	–	–	158	(40, 89, 90, 96, 97, 101, 103–120)
PIK3R1	18/49	2	–	–	–	–	–	64	(94, 95, 98–100, 102, 113, 117, 121–124)
NFKB1	1/15	–	–	2 (*M. avium* complex)	–	–	–	56	(40, 82, 131–137)
NFKB2 DN and GOF	0/11	–	–	1 (*M. kansasii)*	–	–	–	29	(40, 138–148)
**CBM complex**									
CARD11 AR LOF	0/2	–	–	–	–	–	–	5	(150, 153, 154, 157)
CARD11 GOF	3/9	–	–	–	–	–	–	9	(152, 155, 156, 158)
CARD11 DN/LOF	0/4	–	–	–	1	–	–	12	(159, 160)
MALT1	0/4	–	–	–	–	–	–	6	(162–165)
CTLA4	4/14	–	–	–	2	–	–	74	(82, 111, 166, 167, 169–181)
LRBA	16/21	–	–	–	–	–	–	108	(82, 174, 179, 180, 182–184, 186, 187, 190–210)
GATA2	3/357	–	1	54 (24 *M. avium complex; 12 M. kansasii; 3 M. abscessus; 1 M. fortuitum; 1 M. scrofulaceum; 1 M. massiliense; 1 M. chelonae; 1 M. szulgai; 1 M. malmoense; 9 NTM)*	–	–	1	357	(40, 211–214, 216, 217, 220–284)
CARMIL2	4/21	–	–	–	–	–	2	21	(286–289)
PGM3	4/37	–	–	–	–	–	–	37	(291, 292, 295–301)

*^a^Numerator/denominator based on paper’s review and personal communications with corresponding authors*.

*^b^Total number of cases published*.

*AR, autosomal recessive; BCG, Bacille Calmette–Guerin; DN, dominant negative; GOF, gain-of-function; LOF, loss-of-function; NTM, nontuberculous mycobacteria; PIDD, primary immunodeficiency diseases; TB, tuberculosis; STAT1, signal transducer and activator of transcription 1; STAT3, signal transducer and activator of transcription 3; CARD, caspase activation and recruitment domain; MALT1, mucosa-associated lymphoid tissue lymphoma translocation gene 1; LRBA, lipopolysaccharide-responsive Beige-like anchor protein; PGM3, phosphoglucomutase 3; BCL10, B-cell leukemia/lymphoma 10; PIK3CD, phosphoinositide kinase 3 catalytic unit delta; PIK3R1, phosphoinositide kinase 3 regulatory subunit 1; NFKB, nuclear factor kappa-light-enhancer of activated B cells; CBM, CARD11, BCL10, MALT1 complex; CARMIL2, capping protein regulator and myosin 1 linker 2; TTC7A, tetratricopeptide repeat domain 7A*.

*BCL10 and TTC7A-deficient patients are not shown in this table as information on their BCG vaccination status could not be retrieved*.

Overall, our own literature review shows an estimated 8% prevalence of mycobacterial infections among patients carrying *STAT1* GOF mutations. Mycobacterial susceptibility is a classical presentation for LOF *STAT1* mutations (15–17) as decreased STAT1-mediated IFN-γ responses impair immunity against mycobacteria (73). Published data demonstrate that excessive activation of STAT1 also impairs IFN-γ responses and, therefore, increases the risk of infection (27, 38). IFN-γ tachyphylaxis has been proposed as one possible explanation to this effect (27). Tight regulation of STAT1 phosphorylation, and in turn its activity, is likely required to mount a protective response against mycobacteria (73).

## GOF Mutations in Signal Transducer and Activator of Transcription 3 (*STAT3*)

Signal transducer and activator of transcription 3 is another member of the STATs protein family of transcription factors. STAT3 is activated by various cytokines and growth factors and plays critical roles in several cell processes, such as cell growth, differentiation, apoptosis, as well as inflammation and oncogenesis (74). LOF-dominant negative (DN) mutations in *STAT3*, cause autosomal-dominant hyper-IgE syndrome, characterized by CMC, bacterial infections, eczema, and connective tissue abnormalities (75, 76). GOF somatic mutations in STAT3 have been associated with some particular types of lymphoproliferative diseases, being found in 40% of large granular lymphocytic leukemia patients (77).

Heterozygous activating germline mutations in *STAT3* were first described in 2014, in a cohort of five patients presenting early-onset autoimmunity (78). One-year later, two groups simultaneously described 14 additional cases, providing informative data on this PIDD phenotype (79, 80). Early-onset autoimmunity was prominent, alongside short stature and lymphoproliferative disease. Recurrent infections were frequent and two patients had malignancies, one adult patient had Hodgkin lymphoma, and one pediatric patient had a T-cell large granular lymphocytic leukemia (79, 80). Immunologically, most patients showed hypogammaglobulinemia, and also decreased numbers of T regulatory cells (79–81).

A NTM infection was reported in one patient with a germline *STAT3* GOF mutation. The patient had received BCG vaccine as a child without any reported complication. However, at the age of 19 years she developed *Mycobacterium avium* pneumonia, followed by dissemination (*M. avium* was isolated from lymph node, feces, and bone marrow samples). The IL-12/IFN-γ pathway was evaluated and found to be normal. The authors hypothesized that the mycobacterial infection could be due to the lack of plasmacytoid dendritic cells detected in the patient (80). A total of four patients received BCG vaccination without complications [(78–80, 82), and Acknowledgments] (Table 2).

The underlying molecular mechanisms of increased transcriptional activity of STAT3 are not yet fully elucidated (83, 84). Although BCG complications have not been reported in these patients [(78–82, 84–88), and Acknowledgments], the occurrence of disseminated environmental mycobacterial infection in one patient raises awareness (80).

## Activated PI3Kδ Syndrome Type 1 and 2

Activated phosphoinositide 3-kinase δ syndrome (APDS) in an immunodeficiency and immune dysregulation syndrome, first described in 2013 (89, 90). It results from pathological hyper activation of PI3Kδ, a lipid kinase responsible for the conversion of phosphatidylinositol 4,5-bisphosphate (PIP_2_) to the second messenger phosphatidylinositol 3,4,5-trisphosphate (PIP_3_). PI3Kδ is a class IA PI3K, composed of a catalytic (p110δ) and a regulatory subunit (p85). It is predominantly expressed in leukocytes and can be induced by several transmembrane receptors, such as antigen receptors, cytokine receptors, toll-like receptors, and costimulatory molecules. It is important for cell growth, proliferation, motility, and survival (91–93).

Germline heterozygous GOF mutations in *PIK3CD*, that encode the catalytic subunit p110δ, were initially described as the genetic cause of APDS (89, 90). Less than a year later, heterozygous GOF mutations in *PIK3R1*, which encodes the regulatory subunit p85, were identified as a phenocopy of this PIDD, being designated APDS type 2 (94, 95). The vast majority of patients present with recurrent sinopulmonary infections commonly leading to bronchiectasis. Other clinical manifestations are highly variable and comprise herpesvirus infections, autoimmune disease (mainly cytopenias), benign lymphoproliferation, and an increased risk of lymphoma (96–99). Immunologically, CD4+ T cell and B cell lymphopenia, progressive loss of naïve CD4+ and CD8+ T cells, expansion of senescent CD8+ T cells, reduced class-switched memory B cells, and poor antibody responses are common findings. A subset of patients presents with hyper IgM (96–101).

In a cohort study of 36 APDS2 patients, Elkaim et al. reported two cases of persistent local skin lesions at BCG vaccination injection site, out of 17 patients who had been BCG vaccinated (99). We gathered information regarding BCG vaccination history from 32 more APDS2 patients and only 1 received the vaccine [(94, 95, 100, 102), and Acknowledgments]. In total, 2/18 BCG-vaccinated APDS2 patients developed local reactions to the vaccine. Likewise, Coulter et al. reported two additional cases of persistent granulomatous local skin reactions to BCG vaccine in a large cohort study of 53 APDS1 patients (97). Through our literature search we identified another local reaction to BCG in an APDS1 patient (103). Collectively, among 10 APDS1 BCG vaccinated patients, three of them developed local reactions to BCG (Table 2) [(40, 90, 97, 101, 103–109), and Acknowledgments].

The ability to control BCG infection was assessed in one APDS1 patient by Chiriaco et al. Monocyte-derived macrophages from the patient failed to restrict intracellular mycobacterial growth *in vitro*, compared to a healthy control. Treatment with a PI3Kδ inhibitor restored patient’s cells ability to kill BCG, suggesting that normal PI3Kδ activity is important to control BCG infection. Despite the failure *in vitro*, this patient received BCG vaccine without complications (104).

There were no reports of disseminated BCG reactions or other mycobacterial infections in the APDS patients reviewed (40, 89, 90, 94–124).

## Defects Affecting the NF-κB Pathway

Nuclear factor kappa-light-chain-enhancer of activated B cells (NF-κB) represents a protein complex that plays pivotal roles in immune and inflammatory responses, cell development, and survival. It is composed of five transcription factors, NFκB1 (p50/p105), NFκB2 (p52/p100), RelA, RelB, and c-Rel that bind to form homodimers or heterodimers. In resting state these dimers are sequestrated in the cytoplasm, tightly controlled by inhibitory proteins. Once activated, they are released after phosphorylation, ubiquitination, and proteasome degradation of their inhibitors and translocated to the nucleus where they control the transcription of a large set of genes (125, 126).

Mutations that affect the NF-kappa-B inhibitor alpha (NFκBIA or IKBA) or the kinases responsible for its inactivation (IKK-β or IKK-γ/NF-κB essential modifier) are known causes of combined immunodeficiency with increased susceptibility to mycobacteria (11, 127, 128). Several patients carrying these mutations present with disseminated disease after receiving BCG vaccine (129, 130).

More recently, germline mutations in two genes encoding transcription factors of the NF-κB family have been described as new causes of PIDD.

*NFKB1* encodes the transcription factor p50 and its precursor p105, which are activated *via* canonical NF-κB signaling pathway (125, 126). Germline heterozygous mutations in *NFKB1* were first described in 2015 (131). They led to haploinsufficiency of NFκB1 and penetrance was shown to be incomplete. The first symptomatic patients reported presented a common variable immunodeficiency (CVID)-like phenotype, suggesting a primarily B-cell disorder. Hypogammaglobulinemia, recurrent infections, autoimmunity, and lymphoproliferation were common findings (82, 131, 132). Finally, new reports expanded the phenotype of this PIDD, highlighting the occurrence of recurrent EBV infection and EBV-driven lymphoproliferative disease, pointing that this defect likely underlies a combined immunodeficiency (133). More than 50 patients carrying *NFKB1* mutations have been published (40, 82, 131–137). No BCG-related complications have been reported but BCG vaccination history from these patients was not described in the literature (40, 82, 131–137). We had access to vaccination status of 15 patients, of whom only one received BCG vaccine, uneventfully (Table 2) [(40, 82, 135, 137), and Acknowledgments]. Interestingly, one group reported *M. avium intracellulare* infections in two patients who were not BCG vaccinated (82).

The second gene, *NFKB2*, encodes p52 and its precursor p100, the transcription factor that mediates the noncanonical NF-κB signaling pathway (125, 126). Initial reports described an autosomal-dominant inheritance and DN effect (138–140). Patients presented with a phenotype of early-onset CVID (hypogammaglobulinemia, impaired antigen response, recurrent infections) associated with unique autoimmune manifestations, ectodermal dysplasia (alopecia areata or totalis and trachyonychia) and endocrine defects, most notably central adrenal insufficiency (138–140). While humoral immunity was always affected, impairment of T lymphocytes and NK cells varied among published cases (138–142). Neither BCG vaccination status nor vaccine adverse reactions were mentioned in the reports (40, 138–148), however, one patient had *Mycobacterium kansasii* infection (146). In 2017, Kuehn et al., described three additional patients with novel mutations in *NFKB2* that resulted in constitutive NFκB2 activation because of a GOF effect (144). Clinically, these patients presented manifestations consistent with a combined immunodeficiency, such as *Pneumocystis jirovecii* pneumonia and severe viral infections. Of notice, no endocrine, ectodermal, or autoimmune manifestations were found. None of these patients were BCG vaccinated, but one patient received anti-tuberculous treatment due to pulmonary nodules and caseating granulomas in a lung biopsy, although a mycobacterial infection was not documented [(144), and Acknowledgments].

In the canonical NF-κB signaling pathway, defects in a protein complex upstream NF-κB have also been described as new causes of PIDD. This complex is composed of three proteins: B-cell leukemia/lymphoma 10 (BCL10), mucosa-associated lymphoid tissue lymphoma translocation gene 1 (MALT1), both ubiquitously expressed, and a member of the caspase activation and recruitment domain (CARD) protein family, which is expressed in a cell-type specific manner (149, 150). In lymphocytes, CARD11, BCL10, and MALT1 bind to form the CBM signalosome complex, which is responsible for triggering NF-κB activation following antigen binding to either T- or B-cell receptor (151).

Germline mutations in *CARD11* have been reported in 26 patients (150, 152–160) leading to three distinct phenotypes. Patients with biallelic LOF mutations presented with a combined immunodeficiency, similarly to what is seen in other mutations affecting the CBM complex (150, 153, 154, 157). Patients with heterozygous GOF mutations showed a lymphoproliferative condition known as “B cell expansion with NF-KB and T-cell anergy” (BENTA) disease (152). This disease is characterized by childhood-onset polyclonal B-cell lymphocytosis, splenomegaly, recurrent bacterial and viral infections, along with impaired vaccine responses (152, 155, 156, 158). In 2017, two groups identified both LOF and dominant-negative heterozygous defects in *CARD11* causing severe atopy and recurrent infections (159, 160). One of these patients had pulmonary tuberculosis (159). BCG-related complications were not mentioned in the reports and BCG vaccination history was not addressed either (150, 152–160). Three BENTA patients were BCG vaccinated without complications (Table 2) [(152, 155, 156, 158), and Acknowledgments].

Concerning the other members of the CBM complex, BCL10 deficiency has been identified in one patient (161) and MALT1 deficiency in six (162–165). These two defects, together with *CARD11* LOF mutations, share a similar phenotype of combined immunodeficiency without T cell lymphopenia, poor growth, severe infections, and gastrointestinal disease. Despite the limited number of BCL10 and MALT1 deficiency cases reported, we chose to include them in this review as an exception to our inclusion criteria as they all are a constitutive part of the CBM complex, and help us to expand our understanding on BCG reactions related to new immunodeficiencies in general and CBM complex defects in particular. Neither BCG vaccination status nor BCG-related complications were described in any of the reports (161–165). From our direct contact with the corresponding authors, we got information on the vaccination status of four MALT1 deficient patients, none of them were BCG vaccinated (Table 2) [(163, 164), and Acknowledgments].

## Cytotoxic T Lymphocyte Antigen-4 (CTLA-4) and Lipopolysaccharide-Responsive Beige-Like Anchor Protein (LRBA) Deficiencies

CTLA4 haploinsufficiency is an immune dysregulation syndrome characterized by hypogammaglobulinemia, progressive B-cell lymphopenia, autoimmunity, and lymphocytic organ infiltration (166, 167). CTLA-4 is as an inhibitory receptor (168) and, once defective, immune tolerance is disrupted due to impairment of regulatory T cell suppressor function along with an increase of autoreactive B cells, among other immunological abnormalities. It is inherited in an autosomal-dominant manner with incomplete penetrance (166, 167). Seventy-six patients carrying pathogenic mutations in CTLA4 have been published to date (82, 111, 166, 167, 169–181), of whom 62 are symptomatic and 14 asymptomatic mutation carriers due to incomplete disease penetrance. No adverse reactions to BCG vaccine have been reported in their past medical history, although patient’s BCG vaccination status information was not available in the publications. We retrieved information on BCG vaccination status of 14 CTLA4-deficient patients, 4 of whom received BCG vaccine without complications (Table 2) [(82, 166, 170, 174, 177, 179), and Acknowledgments]. Moreover, there were no reports of infections caused by weakly virulent mycobacteria in this cohort (82, 111, 166, 167, 169–181). Two patients did have pulmonary tuberculosis in their early twenties (167). They both progressed to significant lung morbidity, but recurrent bacterial pneumonias also accounted for their unfavorable pulmonary outcomes.

LRBA protein deficiency is closely related to CLTA-4 haploinsufficiency in terms of its pathophysiology (182). It is an autosomal recessive disease with almost complete penetrance, also characterized by recurrent infections, hypogammaglobulinemia, autoimmunity, and lymphocytic organ infiltration (183, 184). Autoimmune disease is particularly severe in the gut, presenting with inflammatory bowel disease-like manifestations (179, 185, 186). LRBA protein protects CTLA-4 from lysosomal degradation, maintaining its intracellular stores (182). Although symptoms are similar to CTLA-4 haploinsufficiency, LRBA deficiency is usually more severe and presents at an earlier age (184, 187). One possible explanation to these differences is that when biallelic mutations of LRBA occur, CTLA-4 surface levels can be even lower than those seen in CTLA-4 haploinsufficiency (188). Also, LRBA protein is present in more cell types than CTLA-4 and not all of its functions are completely known (189). Despite the broader phenotype, over 100 LRBA-deficient patients have been described so far and no adverse reactions to BCG vaccine or any mycobacterial disease have been highlighted in their reports (82, 174, 179, 180, 182–184, 186, 187, 190–210). Out of 21 LRBA-deficient patients that we had access to BCG vaccination history, 16 received BCG vaccine without adverse reactions (Table 2) [(82, 174, 179, 185, 191, 193, 202, 205, 210), and Acknowledgments].

## GATA2 Deficiency

GATA2 deficiency was described as a new PIDD in 2011 (211–214). Heterozygous germline mutations in *GATA2*, a highly pleiotropic gene that encodes the hematopoietic transcription factor GATA2 (215), revealed to be the unifying genetic cause of four apparently distinct syndromes: monocytopenia with *M. avium* complex (MonoMAC) (216), dendritic cell, monocyte, B and NK lymphoid deficiency (DCML) (217), primary lymphedema with myelodysplasia (Emberger syndrome) (218), and familial myelodysplastic syndrome/acute myeloid leukemia (219).

As more patients were described, the phenotype was broadened and significant overlap among previously known syndromes was noticed (220, 221). Childhood-onset cases have been reported (222), but most patients start manifesting symptoms later in life (220, 221). Patients present increased susceptibility to infections, mainly viral (particularly human papillomavirus), mycobacterial, and fungal (220, 221). Lung involvement is common, and a subset of patients present with pulmonary alveolar proteinosis (223, 224). Lymphedema and hearing loss are also common (221, 222). Most patients progress to myelodysplasia and are at increased risk of acute myeloid leukemia (221, 225).

Immunologically, progressive monocytopenia, B and NK lymphocytopenia, and absence of dendritic cells are hallmarks of the disease. CD4+ lymphopenia and neutropenia can also be seen but are less pronounced (220, 221). Bone marrow usually shows multilineage dysplasia and atypical megakaryocytes (221, 226).

Susceptibility to NTM is a remarkable finding in GATA2 deficiency. Fifty-four cases of NTM (212, 213, 216, 217, 220, 221, 224, 227–243) and only one case of disseminated *M. tuberculosis* (244) have been described in more than 350 GATA2-deficient patients published (Table 2) (40, 211–214, 216, 217, 220–284).

Information on BCG vaccination was available only for three patients in the reports (217, 241, 245). While two of them did not experience vaccine-related complications, one developed disseminated BCG infection at the age of 12 years, as the initial presentation of his disease. Four years later, he underwent a successful bone marrow transplantation from a matched unrelated donor (285). In the first month post-transplant, the patient had immune reconstitution syndrome (fever and rash) treated with a short course of systemic corticosteroids. Antimycobacterial treatment was stopped within a year.

## CARMIL2 Deficiency

Recently, biallelic LOF mutations in *RLTPR*, also known as *CARMIL2*, were described as a new PIDD (286–289). Capping protein regulator and myosin 1 linker 2 (CARMIL2) is a cytosolic protein found to be important for CD28-co-stimulation pathway and migration in T cells, as well as BCR-mediated activation of B cells (289, 290).

CARMIL2-deficient patients presented with recurrent bacterial respiratory and cutaneous infections, widespread warts alongside other viral infections (varicella zoster virus, molluscum contagiosum, and EBV), persistent dermatitis (eczema or psoriasiform hyperkeratotic lesions), and CMC. Inflammatory bowel disease and chronic esophagitis were seen in some patients. Four patients had disseminated EBV+ smooth muscle tumors (286–289).

Immunologically, patients shared a significant reduction in regulatory T cells, CD4+ memory and follicular helper cells. Th1 and Th17 cytokine production were impaired. Switched memory B cells counts were low and antibody responses to vaccines were poor (286–289).

Mycobacterial infections were seen in 2 out of 21 published cases. They both had disseminated tuberculosis (described as multifocal disease in one patient and miliary tuberculosis in the other). BCG vaccine had been given to both patients, uneventfully (289). Two other CARMIL2-deficient patients received BCG vaccine, without complications (287) (Table 2). Information on BCG vaccination status of the other CARMIL2-deficient patients was not available (286–289).

## Phosphoglucomutase 3 (PGM3) Deficiency

Recently, biallelic hypomorphic mutations in *PGM3* have been described as a new congenital disorder of glycosylation resulting in PIDD (291, 292). PGM3 is an enzyme that catalyzes the conversion of *N*-acetylglucosamine-6-phosphate (GlcNAc-6-P) to 1-phosphate (GlcNAc-1-P) which is necessary for the generation of uridine diphosphate *N*-acetylglucosamine, an important precursor to multiple glycosylation pathways. Glycosylation is a complex posttranslational enzymatic process responsible for the attachment and trimming of glycans to proteins and lipids, critically affecting their structure and function (293, 294).

Congenital disorders of glycosylation usually manifest with broad, multisystemic symptoms (294). Two distinct phenotypes have been described for *PGM3* mutations, possibly correlated with levels of residual enzymatic activity (291, 292, 295). The majority of patients presented with an AR hyper-IgE syndrome phenotype of eczema and multiple manifestations of atopy, recurrent sinopulmonary and skin infections, failure to thrive, and varying degrees of neurological impairment. Dysmorphic features were present in some patients. Serum IgE levels and eosinophils counts were elevated and T cell lymphopenia was frequent (291, 292, 296). A subset of patients manifested a severe combined immunodeficiency phenotype with profound T- and B-cell lymphopenia but normal IgE levels. Neutropenia was also present. Skeletal dysplasia and multiple dysmorphisms were common findings among these patients (295, 297, 298).

There were no reports of BCG-related complications in the PGM3-deficient patients (291, 292, 295–301). Information on vaccination status was not provided in most reports, including all patients with severe combined immunodeficiency (SCID) presentation. Four patients were BCG vaccinated uneventfully (Table 2) (301).

## TTC7A Deficiency

The hereditary association of multiple intestinal atresia (MIA) and immunodeficiency has been long reported in the literature (302). However, the discovery of mutations in *TTC7A* as its underlying cause was only possible in 2013, after the advent of whole exome sequencing (303, 304).

Tetratricopeptide repeat domain 7A (TTC7A) protein function was not clear until the description of TTC7A-deficient patients (305). To date, more than 50 cases of biallelic mutations in *TTC7A* have been reported (303, 304, 306–317), and this protein was found critical to gut and immune system development and homeostasis (305).

The initial phenotype recognized is shared by the majority of patients reported. MIA requiring surgical interventions is associated with profound lymphopenia, hypogammaglobulinemia, severe and recurrent infections, with high incidence of sepsis caused by intestinal microbes (303, 304, 306–309, 313). Some patients present MIA alone (304, 308), and others manifest very early onset inflammatory bowel disease (204, 307). The prognosis is poor and most patients die at a young age. More recently, a milder phenotype of enteropathy and predominantly humoral immunodeficiency has been reported (315).

None of the reports of TTC7A-deficient patients had information regarding BCG vaccination status. Neither BCG vaccine-related complications nor mycobacterial infections were reported (Table 2) (303, 304, 306–317).

## Conclusion

The prevalence of BCG-associated complications in the general population can vary widely depending on the reporting country, the vaccine strain used, and the age at vaccination. Reports of 1 in 2,500 vaccines presenting with localized BCG-associated complications, and 1 in 100,000 presenting with disseminated complications represent a fair estimate of the general prevalence of such side effects. However, when focused on patients with SCID, the most severe forms of PIDD, ~1 in 2 (51%) develop BCG-associated complications after vaccination, 2/3 presenting with disseminated disease, and the remaining 1/3 as localized disease (9). In a recent cohort of 71 chronic granulomatous disease (CGD) patients, 75% presented with BCG-related complications (318). Moreover, among patients with MSMD due to mutations in IL12Rβ1, ~3 in 4 (77%) present with BCG-associated disease, 4/5 as disseminated and 1/5 as localized complications (319). While these diseases are well known for their increased susceptibility to BCG-related side effects, less specific information is available regarding other or more recently described PIDD.

In this review, we focused on PIDD first reported since 2010. In order to assure a fair patient representation, we limited our analysis to those diseases affecting 20 or more unrelated patients belonging to 10 or more families, with defects impairing areas of the immune system known to be involved in the control of mycobacterial infections.

Not surprisingly and as previously shown by Toubiana et al. (24), we found that patients with GOF STAT1 mutations showed an increased susceptibility to mycobacterial diseases, including BCG. Of note, our analysis of the published data also showed that patients with APDS 1 and 2 appear to have an increased risk for BCG-related localized complications. These findings *per se* can involve actionable recommendations as to ban BCG vaccination in STAT1 GOF, APDS1, and APDS2 patients and in their genetically untested newborn relatives until their status is clarified.

Among other newly described PIDD, no BCG-related complications were reported in the relatively frequent LRBA and CTLA4-deficient patients, or in NFKB1 and NFKB2-mutated individuals. As a limitation of this review, and despite our efforts to try to retrieve as much information as possible regarding BCG vaccination status and complications in these diseases, this negative data have to be taken with a grain of salt as the *n* of vaccinated patients might not have been sufficient to capture low but still relevant BCG complication rates in these diseases.

In summary, despite its debatable efficacy against tuberculosis, BCG remains one of the most popular and with higher coverage rate vaccines in many parts of the world. Interestingly, certain vaccines—BCG included—have been recently suggested to provide a significantly positive impact on overall health through heterologous immunity, sometimes even surpassing the specific protection originally intended (320). In any case, as clinicians taking care of a vulnerable population of individuals with inborn errors of their immune system, we should remain vigilant of any preventable medical interventions that can detrimentally impact our patients. As already well described, BCG vaccination should be avoided not only in SCID, MSMD, and CGD patients (321) but also in other particular newly described PIDD (e.g., STAT1 GOF, APDS1, and APDS2) in which its complication rates have shown to markedly exceed when compared to the general population.

## Author Contributions

CJNS contributed to the research design and wrote the first draft. SDR contributed to the research design and supervised the whole project.

## Conflict of Interest Statement

The authors declare that the research was conducted in the absence of any commercial or financial relationships that could be construed as a potential conflict of interest.
